# Metabolomic and Lipidomic Signatures of Metabolic Syndrome and its Physiological Components in Adults: A Systematic Review

**DOI:** 10.1038/s41598-019-56909-7

**Published:** 2020-01-20

**Authors:** Stéphanie Monnerie, Blandine Comte, Daniela Ziegler, José A. Morais, Estelle Pujos-Guillot, Pierrette Gaudreau

**Affiliations:** 1 Université Clermont Auvergne, INRA, UNH, Mapping, F-63000 Clermont Ferrand, France; 2 Centre Hospitalier de l’Université de Montréal (CHUM), Montréal, Canada; 3 Département de Gériatrie, Université McGill, Montréal, Canada; 4 Université Clermont Auvergne, INRA, UNH, Plateforme d’Exploration du Métabolisme, MetaboHUB Clermont, F-63000 Clermont-Ferrand, France; 5 Centre de Recherche du CHUM, Montréal, Canada; 6 Département de médecine, Université de Montréal, Montréal, Canada

**Keywords:** Metabolomics, Biomarkers

## Abstract

The aim of this work was to conduct a systematic review of human studies on metabolite/lipid biomarkers of metabolic syndrome (MetS) and its components, and provide recommendations for future studies. The search was performed in MEDLINE, EMBASE, EMB Review, CINHAL Complete, PubMed, and on grey literature, for population studies identifying MetS biomarkers from metabolomics/lipidomics. Extracted data included population, design, number of subjects, sex/gender, clinical characteristics and main outcome. Data were collected regarding biological samples, analytical methods, and statistics. Metabolites were compiled by biochemical families including listings of their significant modulations. Finally, results from the different studies were compared. The search yielded 31 eligible studies (2005–2019). A first category of articles identified prevalent and incident MetS biomarkers using mainly targeted metabolomics. Even though the population characteristics were quite homogeneous, results were difficult to compare in terms of modulated metabolites because of the lack of methodological standardization. A second category, focusing on MetS components, allowed comparing more than 300 metabolites, mainly associated with the glycemic component. Finally, this review included also publications studying type 2 diabetes as a whole set of metabolic risks, raising the interest of reporting metabolomics/lipidomics signatures to reflect the metabolic phenotypic spectrum in systems approaches.

## Introduction

Metabolic syndrome (MetS) is a complex health condition responsible for the concurrence of several metabolic abnormalities and cardiovascular disturbances. Despite a lack of unified definition among health organizations (*e.g*. National Cholesterol Education Program (NCEP), International Diabetes Federation (IDF), World Health Organization (WHO)), MetS comprises glucose metabolism dysregulation due to insulin resistance, central obesity, dyslipidemia, including increased blood triglycerides (TG) and decreased high-density lipoprotein cholesterol (HDL-C), and hypertension^1–4^. This combination of risk factors favor adverse outcomes such as type 2 diabetes (T2D) and cardiovascular disease (CVD) and increased mortality rate by approximately 1.5-fold^5^. It is generally accepted that the prevalence of MetS is on the rise in accordance with increasing body mass index (BMI) and aging of the population^6^. Because several clinical definitions co-exist, the true prevalence of MetS is difficult to establish. In spite of this, U.S. surveys indicate that one-third of adults^7–9^, including young adults^10^ have MetS. Moreover, by the age of 60, the prevalence reaches 42% compared to 7% for young adults^11^. Europe has not been spared from such epidemic, with also a sharp increase of MetS among older adults^12^. Therefore, it is now accepted that MetS represents a global p ublic health concern with a worldwide prevalence ranging from 10 to 84%, depending on the ethnicity, age and sex/gender^13,14^.

MetS is recognized as a progressive pathophysiological state, being part of the trajectory leading to pre-diabetes, T2D and CVD^15^. I n fact, MetS is not only a precursor but also a predictor of T2D development^16–19^. Risks of adverse health outcomes increase substantially with accumulation of MetS clinical components and deleterious environmental factors (*e.g*. inactivity, Western-type diet). In this context, it is important to better characterize intermediate phenotypes associated with metabolic abnormalities. Biomarkers are considered useful to disentangle the exposure-disease relationships in chronic metabolic disorders and provide sensitive tools for a better identification and stratification of high-risk individuals^20^. Timely identification of MetS physiological disturbances should allow pinpointing individuals at highest risk to develop T2D, CVD, and multi-organ damage. Moreover, studies of their trajectories should provide insights into key periods for lifestyle intervention, risk factor management, and robustness of pharmacological treatment.

Over the last few years, omics technologies allowed obtaining an integrated view of biological systems, bridging the genotype-to-phenotype gap using a systems biology approach to better define the phenotype. In chronic metabolic diseases, the phenotype is complex and dynamic, because of the occurrence of multiple interactions among genetic and environmental factors^21^. In this setting, metabolomics, introduced by Nicholson *et al*. 1999^22^, aiming at measuring all small molecules/metabolites present in a biological system and accessible to analysis, represents a powerful phenotyping tool. Indeed, it provides metabolic profiles that represent an integrated view of metabolism because it allows a sensitive detection of molecular changes over time, resulting from the interaction between intrinsic and extrinsic factors^23^. Metabolites, used as single targets or in combination within a comprehensive signature, are thus promising biomarkers to reveal early metabolic dysfunctions, when conventional clinical markers have a limited ability for risk assessment and stratification. Metabolomics has therefore been widely applied for metabolic disease diagnosis and candidate biomarker discovery as well as pathophysiological exploration of underlying mechanisms, and prognosis and prediction^24,25^.

Because the human metabolome is complex (*e.g*. large concentration ranges, high number of metabolites, chemical diversity), different analytical strategies and methods have been developed. The approach can be untargeted, as a data driven approach dedicated to biomarker discovery, or targeted when it is focused on the detection and quantification of specific classes of compounds, or subsets of known metabolic pathways^26^. For example, lipidomics has been described as a subsection of metabolomics dedicated to lipid analysis, even if there is a continuum of polarity between lipophilic and hydrophilic metabolites^27^. To cover this wide diversity of metabolites present in a given biological sample, diverse analytical platforms are used. Mass Spectrometry (MS) coupled with gas or liquid chromatography (GC- or LC-, respectively) and Nuclear Magnetic Resonance (NMR) Spectroscopy are the two main analytical techniques used. NMR is non-destructive, rapid, and highly robust, which is convenient for a rapid screening of biological sample^28^ but suffers from limited sensitivity (less than 100 metabolites in most biological samples by current methods). Advances in MS and its hyphenated techniques, particularly the increase of their respective resolving and separation powers, significantly impacted metabolomics research allowing for higher sensitivity and broader metabolome coverage^29^. Nonetheless, these MS-based techniques still lack standardization and throughput. In addition, technical factors (time of sampling, sample type, stability) have to be considered for metabolome investigations and the results of different studies need to be compared. Interestingly, certified commercial targeted LC-MS based assays or platforms became available during the last years (*e.g*. Biocrates, Metabolon).

Considering the diversity of experimental design and analytical methods to characterize the multifaceted physiopathology of MetS, it is necessary to rigorously analyse the scientific literature to answer the general question “Do metabolomic/lipidomic profiles of MetS and/or its clinical components allow distinguishing from healthy individuals and do they expand the current knowledge about MetS phenotypes?”. The aim of this work was therefore to conduct a systematic review of human studies on metabolite/lipid markers of MetS and its individual clinical components and provide recommendations for improving the experimental design and result reports of MetS biomarkers.

## Results

### Search results

**Figure 1:**
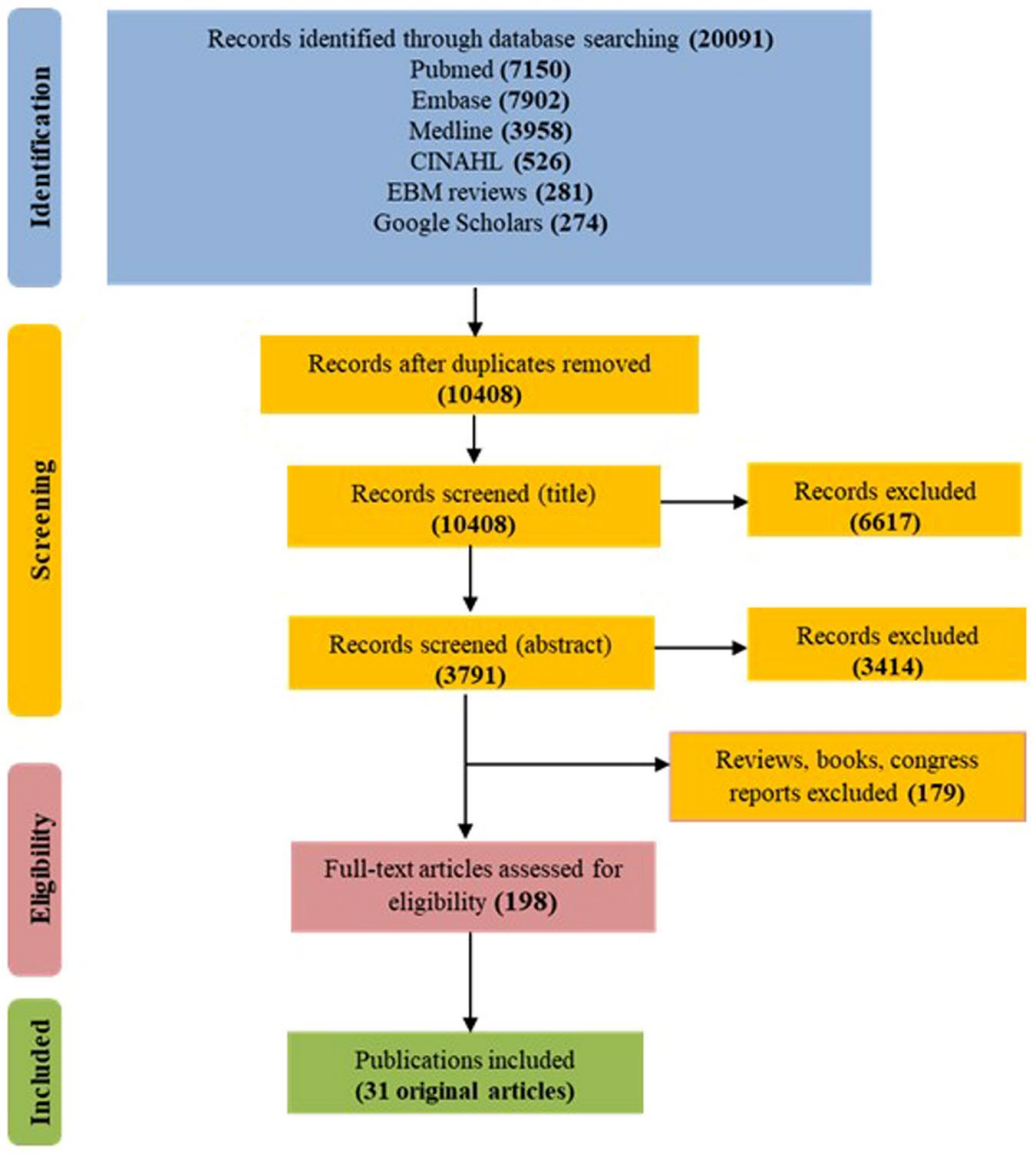
Flow diagram of reviewed citations modified from PRISMA flow diagram 2009^61^.

The primary search identified 20,091 records from five databases (Fig. 1). After removing duplicates, 10,408 original publications were screened for titles and abstracts. Following title screening, 6617 of them were discarded and an additional 3414 were excluded after reading the abstracts, in accordance with the identified inclusion/exclusion criteria. Among the 377 remaining articles, 97 were excluded because they were reviews and 82 more because they were books, congress reports and proceedings. Finally, the full content of 198 original articles was read and analysed for eligibility by three independent authors, and 31 of them were retained for the present review.

These articles were published between 2005 and 2019, 30 out of 31 were published over the last 7 years, and 19 since 2016. Three categories of articles were identified, depending on the main outcome and study design, and the same article could be classified in more than one category depending on the approaches. Twelve of them were case/control studies on MetS with the objective of identifying prevalent or incident MetS biomarkers (Table 1). Sixteen were focussing on MetS components and studied the correlations/associations between identified metabolites and MetS criteria (Table 2). Finally, four articles identified prevalent T2D biomarkers (Table 3) and four others were prospective studies of associations between metabolites and incident T2D (Table 4).

**Table 1:** Characteristics of case/control studies on MetS.

Reference (Study, population location)	Study design	Outcome (MetS definition)	N	Age range	Gender	Population sample characteristics								
						N	Type	Age	BMI	WC (cm)	Sys BP / Dia BP (mmHg)	Glucose (mM)	TG (mM)	HDL-C (mM)
Caimi_2012^63^ (Italy)	Case/Control	MetS (IDF) + T2D (IDF)	160	—	M + W	106	MetS	54 ± 9	32 ± 5	107 ± 11	132 ± 16 / 81 ± 10	6.3 ± 2.5	2.5 ± 1.7	1.0 ± 0.3
						54	non-MetS	No population description						
Capel_2018^30^ (Mona Lisa survey, France)	Case/Control	MetS (Alberti 2009)	298	35–74	M + W	61	MetS	54 ± 8	30 ± 5	102 ± 10	141 ± 20 / 88 ± 12	5.7 ± 0.6	2.0 ± 0.8	1.3 ± 0.3
						237	non-MetS	48 ± 8	24 ± 3	85 ± 10	122 ± 16 / 77 ± 10	5.1 ± 0.4	1.0 ± 0.4	1.6 ± 0.3
James-Todd_2016^64^ (NHANES, USA)	Case/Control	MetS (NCEP ATP III)	1338	20–80	M	464	MetS	52 ± 22	33 ± 7	114 ± 22	129 ± 22 / 74 ± 22	6.7 ± 4.3	2.8 ± 4.3	1.1 ± 0.4
						924	non-MetS	43 ± 30	27 ± 6	96 ± 30	119 ± 30 / 70 ± 30	5.6 ± 1.2	1.4 ± 0.9	1.1 ± 0.6
	Case/Control	MetS (NCEP ATP III)	1331	20–81	W	501	MetS	53 ± 22	33 ± 9	107 ± 22	126 ± 22 / 71 ± 22	6.4 ± 2.2	2.1 ± 2.2	1.3 ± 0.5
						830	non-MetS	43 ± 29	27 ± 6	89 ± 29	115 ± 29 / 69 ± 12	5.1 ± 5.8	1.1 ± 0.9	1.6 ± 0.3
Kulkarni_2013^65^ (SAFHS, USA)	Case/Control	MetS (IDF)	1358	22–56	M + W	1358	total pop	39 ± 17	29 ± 7	95 ± 17	120 ± 19 /71 ± 10	5.6 ± 2.5	1.7 ± 1.2	1.3 ± 0.3
Ntzouvani_2017^66^ (Greece)	Case/Control	MetS (IDF)	100	over 30	M	56	MetS	58* (47;64)	29* (27;32)	105* (100;112)	134* (126;138) / 85*(79;90)	5.5* (5.0; 6.1)	1.9* (1.4;2.5)	1.0* (0.9;1.2)
						44	non-MetS	54* (47;57)	25* (24;27)	91* (87;93)	124* (116;131) / 80*(71;86)	5.1* (4.8; 5.4)	1.1* (0.8;1.4)	1.3* (1.1;1.5)
Olszanecka_2016^67^ (Poland)	Case/Control	MetS (IDF)	152	40–60	W	63	MetS	51 ± 3	29 ± 3	90 ± 7	163 ± 20 / 93 ± 12	5.3 ± 0.6	2.3 ± 1.2	1.3 ± 0.3
						89	non-MetS	51 ± 2	26 ± 3	84 ± 8	151 ± 13 / 89 ± 11	4.9 ± 0.4	1.2 ± 0.8	1.7 ± 0.3
Ramakrishanan_2018^68^ (USA)	Case/Control	MetS (NCEP ATP III)	50	24–72	M + W	30	MetS	53 ± 9	35 ± 6	109 ± 14	132 ± 11 / 80 ± 9	5.4 ± 0.7	1.7	1.0 ± 0.3
						20	non-MetS	48 ± 13	30 ± 6	92 ± 14	117 ± 12 / 14 ± 9	4.8 ± 0.4	0.7	1.3 ± 0.3
Shim_2019^69^ (USA)	Case/Control	MetS (NCEP ATP III)	50	24–72	M + W	30	MetS	53 ± 9	35 ± 6	109 ± 14	132 ± 11 / 80 ± 9	5.4 ± 0.7	1.7	1.0 ± 0.3
						20	non-MetS	48 ± 13	30 ± 6	92 ± 14	117 ± 12 / 14 ± 9	4.8 ± 0.4	0.7	1.3 ± 0.3
Surowiec_2018^31^ (Leiden Longevity Study, Netherlands)	Case/Control	MetS (NCEP ATP III)	115	—	M + W	50	MetS	64 ± 6	NA	106 ± 10	147 ± 18 / 85 ± 9	6.9 ± 3	2.3 ± 1.3	1.1 ± 0.3
						65	non-MetS	62 ± 7	NA	96 ± 12	130 ± 18 / 77 ± 9	5.4 ± 1.3	1.2 ± 0.5	1.6 ± 0.4
Tremblay-Franco_2015^70^ (Finland)	Case/Control	MetS (NCEP ATP III) + obesity	285	around 40	M + W	75	MetS	46 ± 10	35 ± 6	NA	135 ± 14 / 87 ± 9	NA	1.6 ± 0.8	1.2 ± 0.3
						210	non-MetS	42 ± 11	25 ± 2	NA	120 ± 12 / 78 ± 8	NA	1.0 ± 0.4	1.5 ± 0.4
Wiklund_2014^57^ (EWI-study, Finland)	Case/Control	MetS (Alberti 2009)	78	around 40	W	36	MetS	44 ± 6	31 ± 3	99 ± 6	136 ± 11 / 84 ± 7	5.5 ± 0.7	2.0 ± 0.9	1.4 ± 0.3
						42	non-MetS	40 ± 8	29 ± 3	96 ± 9	122 ± 7 / 78 ± 6	5.1 ± 0.3	1.0 ± 0.3	1.6 ± 0.3
Antonio_2015^71^ (EMAS, Europe)	Prospective (4 years follow-up)	MetS (NCEP ATP III) prediction	1651	40–79	M	289	MetS	59 ± 10	28 ± 3	101 ± 8	147 ± 21 / 88 ± 13	5.5 ± 1.0	1.5 ± 0.8	1.4 ± 0.4
						1362	non-MetS	59 ± 11	26 ± 3	93 ± 9	142 ± 20 / 85 ± 11	5.3 ± 0.8	1.2 ± 0.6	1.5 ± 0.4
Pujos-Guillot_2017^58^ (GAZEL, France)	Prospective (5 years follow-up)	MetS (NCEP ATP III) prediction	112	52–64	M	56	MetS	59 ± 3	27 ± 1	95 ± 4	137 ± 14 / 80 ± 8	6.6 ± 1.3	1.2 ± 0.5	1.5 ± 0.3
						56	non-MetS	59 ± 3	27 ± 1	92 ± 5	129 ± 12 / 78 ± 8	5.5 ± 0.5	1.0 ± 0.4	1.5 ± 0.4

BMI = body mass index; WC = waist circumference; BP = blood pressure (sys = systolic; dia = diastolic); TG = triglycerides; HDL-C = high-density lipoprotein cholesterol. Mean values ± SD; *Median value (25th; 75th percentiles).

**Table 2:** Characteristics of studies investigating correlations between metabolites and MetS criteria.

Reference (Study, population location)	Study design	Outcome (definition)	N	Age range	Gender	Population sample characteristics							
						Mean type (available or calculated)	Age	BMI	WC (cm)	Sys BP /Dia BP (mmHg)	Glucose (mM)	TG (mM)	HDL-C (mM)
Barrea_2018^72^ (Italy)	—	MetS (NCEP ATP III)	137	20–63	M + W	Calculated	36	33	109	126 / 80	5.5	1.6	1.1
Blouin_2005^73^ (Quebec family study (QFS), Quebec (CAN))	—	MetS (NCEP ATP III)	130	20–71	M	Available	43 ± 15	27 ± 5	93 ± 14	117 ± 16 / 73 ± 10	5.5 ± 1.1	1.5 ± 0.8	1.1 ± 0.3
Caimi_2012^63^ (Italy)	Case/Control	MetS ± T2D (IDF)	160	—	M + W	All MetS	54 ± 9	32 ± 5	107 ± 11	132 ± 16 / 81 ± 10	6.3 ± 2.5	2.5 ± 1.7	1.0 ± 0.3
Cheng_2012^74^ (Framingham Heart Study (FHS), USA) (Malmö Diet and Cancer Study (MDC), Sweden)	Case/Control	Cardio-metabolic risk	1015	47–65	M + W	Available	56 ± 9	28 ± 5	96 ± 14	129 ± 18 / 76 ± 10	5.4 ± 0.6	1.8 ± 1.2	1.2 ± 0.4
	Case/Control	Cardio-metabolic risk	746	53–65	M + W	Available	59 ± 6	27 ± 4	88 ± 13	147 ± 19 / 90 ± 9	5.1 ± 0.5	1.3 ± NA	1.3 ± 0.3
Favennec_2015^75^ (D.E.S.I.R. cohort, France)	Case/Control	T2D	1048	37–60	M + W	Calculated	48	25	85	NA	5.5	NA	NA
(Biological Atlas of Severe Obesity (ABOS), France)	Case/Control	Obesity	109	26–56	W	Calculated	46	25	121	NA	6.6	NA	NA
Gao_2019^76^ (CODING, Canada)	—	MetS	536	—	M	Available	42 ± 13	28 ± 5	99 ± 13	133 ± 15 / 84 ± 10	5.3 ± 0.7	1.5 ± 1	1.2 ± 0.3
			545	—	W	Available	45 ± 11	27 ± 5	91 ± 15	123 ± 16 / 80 ± 11	5.1 ± 0.7	1.2 ± 0.7	1.5 ± 0.4
Ho_2016^77^ (Framingham Heart Study (FHS), USA)	—	BMI	2383	45–65	M + W	Available	55 ± 10	28 ± 5	NA	126 ± 19 75 ± 10	5.3* (4.9;5.7)	1.4* (1.0;2.0)	1.2* (1.0;1.5)
Huynh_2019^78^ (AusDiab, Australia)	—	Cardio-metabolic risk	389	—	M + W	Available	55 ± 12	27 ± 4	NA	131 ± 18 / 71 ± 11	5.3 ± 0.4	1.5 ± 0.9	1.46 ± 0.4
Liu_2017^79^ (ERF, Netherlands)	Case/Control	T2D	2776	—	M + W	Calculated	49	27	NA	140 / 80	4.7	1.2	1.3
Marchand_2018^80^ (Quebec (CAN))	—	Insulin resistance	101	48–68	W	Available	57 ± 4	28 ± 5	89 ± 12	130 ± 15 / 82 ± 7	5.6 ± 0.8	1.3 ± 0.7	1.4 ± 0.4
Neeland_2018^81^ (DHS, USA)	—	T2D	3072	18–65	M + W	Available	43 ± 10	28	NA	119 / NA	5	5.2	2.7
Ntzouvani_2017^66^ (Greece)	Case/Control	MetS (IDF)	100	over 30	M	Calculated	56	27	NA	130 / 83	5.3	1.5	1.1
Ottosson_2018^82^ (Malmö Preventive Project, Sweden)	—	T2D	1084	—	M + W	Calculated	69	27	NA	147 / NA	5.5	1.3	1.3
Ramakrishanan_2018^68^ (USA)	Case/Control	MetS (NCEP ATP III)	50	24–72	M + W	Calculated	51	33	102	126 / 78	5.2	1.3	1.2
Shim_2019^69^ (USA)	Case/Control	MetS (NCEP ATP III)	50	24–72	M + W	Calculated	51	33	102	126 / 78	5.2	1.3	1.2
Wang-Satler_2012^83^ (KORA, Germany)	Case/Control	T2D	1297	58–72	M + W	Calculated	64	28	NA	135 / NA	5.6	1.5	1.5

BMI = body mass index; WC = waist circumference; BP = blood pressure (sys = systolic; dia = diastolic); TG = triglycerides; HDL-C = high-density lipoprotein cholesterol; NA = not available; ‘Calculated mean type’ refers to clinical variable means that were calculated, when missing, from the available data in the publication. Mean values ± SD; *Median value (25th; 7 = th percentiles).

**Table 3:** Characteristics of case/control studies on T2D.

Reference (Study, population location)	Study design	Outcome	N	Age range	Gender	Population sample characteristics									Methods			Results
						N	Type	Age	BMI	WC (cm)	Sys BP Dia BP (mmHg)	Glucose (mM)	TG (mM)	HDL-C (mM)	Biological fluid / sample	Data production	Statistical method (covariates in fully adjusted model)	Family with significantly modulated metabolites
Lind_2012^84^ (PIVUS, Sweden)	Case/Control	T2D	1016	70	M+W	119	T2D	70	29±5	98±11	155±24 80±12	8.4±3.1	1.5±0.8	1.4±0.4	Serum / NA	Targeted LC/MS metabolomics	Logistic regression (Sex/gender, serum cholesterol and TG, BMI, smoking and exercise habits, educational levels)	Phtalates
						897	non-T2D	70	27±4	90±11	149±22 79±10	4.9±0.5	1.3±0.6	1.5±0.4				
Liu_2017^85^ (ERF, Netherland)	Case/Control	T2D	2776	48–60	M+W	212	T2D	60±12	30±6	99±14	154±21 83±10	7.4±2.2	1.6* (1.1;1.9)	1.1±0.3	Plasma / lipid extract + plasma	Targeted LC/MS-MS + NMR lipidomics and metabolomics	Logistic regression (Age, sex/gender and lipid-lowering medication)	Amino acids and derivatives, carbohydrates and derivatives, cholesterol and oxysterols, glycerolipids, glycerophospholipids
						2564	non-T2D	48±14	27±5	87±13	139±20 80±10	4.5±0.7	1.2* (0.8;1.6)	1.3±0.4				Glycolysis related metabolites, organic acids, peptides
Meikle_2013^86^ (AusDiab, Australia)	Case/Control	T2D	287	52–73	M+W	117	T2D	62* (52;73)	28* (26;31)	97* (89;104)	143*(131;154) NA	6.9* (5.7; 7.4)	1.9* (1.3; 2.9)	1.2* (1.0;1.5)	Plasma / lipid fraction	Targeted LC/MS lipidomics	Logistic regression (Age, sex/gender, WC and SBP) BH corrected p-value <0.05	Ceramides, cholesterol and oxysterols, glycerolipids, glycerophospholipids
						170	non-T2D	60* (49;72)	26* (24;28)	90* (83; 98)	133*(121;146) NA	5.3* (5.1;5.6)	1.2* (0.9;1.6)	1.4* (1.2;1.7)				
Wang-Satler_2012^83^ (KORA, Germany)	Case/Control	T2D	957	58–72	M+W	91	T2D	66±5	30±4	NA	147±22 NA	7.4±1.8	1.9±1.2	1.3±0.4	Serum / serum	Targeted LC/MS metabolomics (AbsoluteIDQ® p180 kit: Biocrates)	Logistic regression (Age, sex/gender, BMI, physical activity, alcohol intake, smoking, SBP and HDL-C + fasting glucose)	Amino acids and derivatives, carbohydrates andderivatives, glycerophospholipids
						866	non-T2D	64±6	28±4	NA	132±19 NA	5.3±0.4	1.4±0.8	1.6±0.4				

BMI = body mass index; WC = waist circumference; BP = blood pressure (sys = systolic; dia = diastolic); TG = triglycerides; HDL-C = high-density lipoprotein cholesterol. Mean values ± SD; *Median value (25^th^; 75^th^ percentiles)

‘Extract’ refers to direct protein precipitation/extraction on raw biological materials; ‘fraction’ refers a separation of biological materials into polar and lipid fractions.

**Table 4:** Characteristics of prospective studies on T2D.

Reference (Study, population location)	Study design	Follow-up time (years)	Outcome	N	Age range	Gender	Population sample characteristics									Methods			Results
							N	Type	Age	BMI	WC (cm)	Sys BP Dia BP (mmHg)	Glucose (mM)	TG (mM)	HDL-C (mM)	Biological fluid / sample	Data production	Statistical method (covariates in fully adjusted model)	Family with significantly modulated metabolites
Peddinti_2017^87^ (Botnia, Finland + DESIR, France)	Case/ Control	10	T2D prediction	543	48–52	M+W	146	T2D	52±1	29±0.4	96±1	139±2 84±1	5.9±0.05	1.7±0.08	1.3±0.03	Plasma / MeOH extract	Semi-targeted LC/MS + GC/MS (Metabolon® platform) metabolomics	Conditional logistic regression FDR q<0.05 (Age, sex/gender, BMI, fasting glucose level and family history of T2D) p- values <0.05 multivariate logistic regression	Amino acids and derivatives, bilirubins, carbohydrates and derivatives, fatty acids and derivatives, quinones and hydroquinones
							397	non-T2D	48±1	26±0.2	88±1	130±1 79±1	5.6±0.03	1.3±0.04	1.4±0.01				
Suvitaival_2017^88^ (METSIM (discovery set), Denmark)	Case/ Control	5	T2D prediction	323	53–65	M	107	T2D	59±6	29±4	102±0	143±16 90±9	6.0±0.5	1.9±1.2	1.3±0.4	Plasma / lipid fraction	Non-targeted LC/MS lipidomics	Logistic regression Model (Age and BMI)	Glycerolipids, glycerophos-pholipids
							216	non-T2D	60±5	26±2	95±7	133±15 85±9	5.2±0.2	1.1±0.5	1.5±0.4				
Wang-Satler_2012^83^ (KORA, Germany)	Case/Control	10	T2D prediction	876	58–72	M+W	91	T2D	66±5	30±4	NA	138±19 NA	5.9±0.6	1.7±0.8	1.3±0.3	Serum / serum	Targeted LC/MS metabolomics (AbsoluteIDQ® p180 kit: Biocrates)	Logistic regression (Age, sex/gender, BMI, physical activity, alcohol intake, smoking, SBP, HDL cholesterol Hb1Ac, fasting glucose and fasting insulin)	Glycerophos-pholipids
							785	non-T2D	63±5	28±4	NA	132±19 NA	5.4±0.5	1.4±0.8	1.6±0.4				
Yengo_2016^89^ (DESIR, Europe)	Case/ Control	9	T2D prediction (ADA)	1067	37–60	M+W	231	T2D	51±9	28±4	94±11	139±17 84±9	5.9±0.6	1.7±1.2	1.5±0.4	Plasma / MeOH extract	Semi-targeted LC/MS-MS + GC/MS (Metabolon® platform) metabolomics	Logistic and Cox regressions	Amino acids and derivatives, carbohydrates and derivatives, carnitines, fatty acids and derivatives, glycerolipids, glycerophos-pholipids, peptides, purines and derivatives, steroids
							836	non-T2D	47±10	25±4	83±11	131±16 80±10	5.3±0.7	1.1±0.7	1.6±0.4				

BMI = body mass index; WC = waist circumference; BP = blood pressure (sys = systolic; dia = diastolic); TG = triglycerides; HDL-C = high-density lipoprotein cholesterol.

‘Extract’ refers to direct protein precipitation/extraction on raw biological materials; ‘fraction’ refers a separation of biological materials into polar and lipid fractions. MeOH: methanol.

### MetS biomarkers: results from case/control studies

Sixteen articles were included in the first section of the systematic review, on prevalent MetS biomarkers identified in case/control studies (Table 1). They provide population characteristics. Most of the studies were performed in populations aged between 40 and 60 years. Generally, MetS cases exhibited three criteria: a high WC combined with two of the following, high glucose, high TG or hypertension. They were compared with healthy controls. These sixteen articles described 409 different modulated metabolites in blood or urine, each one discriminating MetS patients and controls from a single studied population for the discovery (Supplemental Table 1a). Ninety of them are amino acids and derivatives, 90 others, di- and tri-glycerides, and around 70 glycerophospholipids. No replication/validation was performed and these biomarkers were mostly identified using targeted MS metabolomics or lipidomics. The metabolites are presented in Supplemental Table 1a with associated references and classified by metabolite families and direction of variation (*i.e*. positive or negative), as well as analytical methods for metabolomics/lipidomics and used statistical parameters/cofactors. A total of twenty-four different metabolites families were found to be involved. The main classes are amino acids and derivatives, carbohydrates and derivatives, glycolysis related metabolites, glycerophospholipids, glycerolipids, sphingolipids, fatty acids, cholesterol and oxysterols, steroids, and peptides.

Two other publications described biomarkers of incident MetS in prospective studies including only men. Nineteen metabolites were identified as belonging to the following chemical families: amino acids and derivatives, carbohydrates and derivatives, carnitines, fatty acids and derivatives, glycerophospholipids, peptides and steroids (Supplemental Table 1b). It is noteworthy that seven among these metabolites were already described as markers of prevalent MetS, namely alanine, glutamic acid, phenylalanine, tyrosine, oleic acid, total and free testosterone.

### Metabolites associated with MetS clinical components

Sixteen articles were included in the second section of the systematic review and are presented in Table 2. In these publications, the main outcome was not only MetS, but also associated components (*e.g*. obesity, cardio-metabolic risk). Each study correlated metabolites and MetS criteria using different statistical approaches (Spearman/Pearson correlations or linear regression). In terms of clinical characteristics, data were generally provided regarding the whole studied populations and therefore are quite heterogeneous within the age range of 36 to 69 years and BMI of 25 to 33 kg/m^2^.

**Figure 2:**
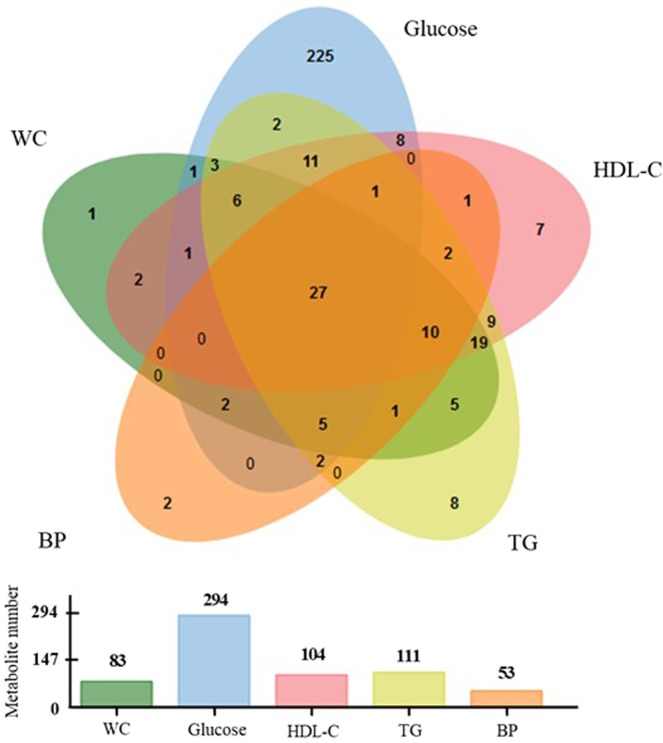
Venn diagram showing the number of metabolites significantly correlated with MetS components, together with respective histogram representing the number of significant metabolites for each clinical MetS components. WC = waist circumference; BP = blood pressure; TG = triglycerides; HDL-C = high-density lipoprotein cholesterol.

Over three hundred metabolites (361) were described as being significantly correlated with one or several MetS criteria, independently (Supplemental Table 2), including 22 metabolite families. Twenty seven of them are correlated with all MetS components (Fig. 2): alanine, choline, glutamate, glutamine, glutamine/glutamate ratio, glycine, isoleucine, L-carnitine, leucine, methionine, phenylalanine, proline, tyrosine, valine, glycerol, 9 TGs, testosterone, alpha-hydroxybutyric acid, and Cer(20:3). Of interest, nineteen of them have already been reported to be prevalent MetS biomarkers in case/control studies (alanine, L-carnitine, choline, glutamate, glutamine, isoleucine, leucine, phenylalanine, proline, tyrosine, valine, and 8 TGs).

Around 10% of the metabolites were common to three of the MetS criteria (all combinations of them). More specifically, about 60% of the identified metabolites showed levels correlated with HDL-C, TG, and glycemia criteria. In addition, this review highlights that some metabolite levels were found to be specifically correlated to each of the MetS criteria (Supplemental Table 3). Seventeen of them were previously described as prevalent MetS biomarkers: 3-hydroxybutyrate, nitric oxides, 5 phospholipids, and 10 TGs.

### The glycemic component: towards T2D

Considering that MetS can lead to T2D and was included in some criteria definition (IDF), we also analyzed articles highlighting an association between prevalent and incident T2D and metabolite dysregulations. A large body of literature was found regarding the investigation of T2D using metabolomics. However, we only selected publications including available clinical data about MetS criteria. Four original articles were selected with case/control design aiming at identifying prevalent T2D markers (Table 3). Four other prospective studies have assessed metabolites associated with incident T2D (Table 4). All these studies have included hypertensive older adults (48 to 70 years) with some cases having a BMI around 30 compared to controls (BMI around 27). Fifty-two metabolites were positively modulated with prevalent T2D from 10 different metabolite families (Supplemental Table 4), identified using targeted MS approaches, predominantly, performed on plasma or serum. The incident markers of T2D were more frequently investigated using un- or semi-targeted MS approaches and were validated within a replication study in different cohorts, revealing 39 modulated blood metabolites (Supplemental Table 5) from 11 chemical families. Of particular interest, three studies used multivariate statistical analyses to define a metabolic signature of T2D-related early metabolic disturbances. Among the individual markers, only isoleucine was already reported as a marker of prevalent T2D.

**Figure 3:**
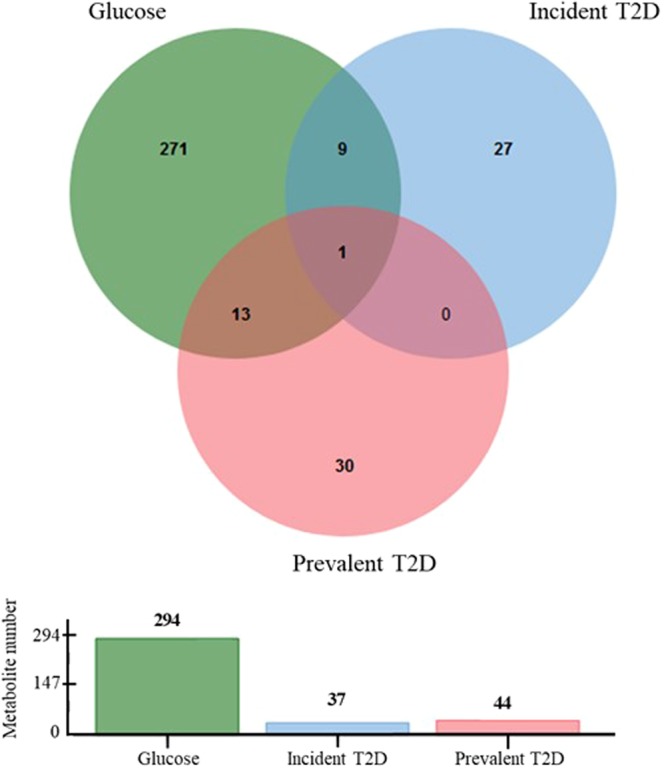
Venn diagram showing the numbers of metabolites significantly modulated with prevalent and incident T2D and the number of metabolites associated with glycemia, together with respective histogram representing the number of significant metabolites for each outcome.

The prevalent and incident T2D markers were then compared to those previously described as being associated with the glucose component (Fig. 3). Thirteen metabolites (mostly amino acids, total hexoses and lipid derivatives) are shared by the prevalent T2D and the glucose component whereas 9 metabolites (mostly amino acids) are shared by the incident T2D and the glucose component of MetS. Of particular interest, the amino acid isoleucine is the only shared metabolite by all these glycemic states.

## Discussion

### MetS biomarkers: results from case/control studies

In the present systematic review, a first category of publications identified prevalent MetS biomarkers in adults using mainly targeted metabolomics approaches. Even if the population characteristics were clearly presented and quite homogeneous, results were difficult to compare in terms of modulated metabolites because of the limited metabolome detected by each single targeted analytical method. However, if the same samples were subjected to different complementary analyses or techniques, some additional metabolites would have been detected. This point is highlighted in two included recent publications that performed semi-targeted approaches that allowed identifying hundreds of modulated metabolites^30,31^. This comparison of throughput and coverage in targeted and non-targeted metabolomics have extensively been discussed in the literature, showing the interest of using multi-platform approaches^32–34^ to obtain a broader scope of the metabolome related to specific phenotypes. However, due to the high costs of analyses, limited biofluid sample volumes and complexity of resulting data treatments, this strategy is still not a current practice.

Because of the targeted aspects of most of the methods, the underlying mechanisms were not explored, and the frequencies of occurrence of specific metabolites described as MetS biomarkers in these studies were low, and not representative of the importance of these metabolites in the physiopathology but can just be related to the choice of the analytical methods.

### Metabolites associated with MetS clinical components

The second category of articles focusing on MetS individual components allowed us comparing metabolites associated with clinical data defining MetS. Amino acids, glycerolipids and glycerophospholipids are the major metabolite classes reported as being correlated. Among lipid species, results were particularly difficult to report and to compare, due to the diversity in notations of lipid structures. In fact, even if several consortia proposed guidelines^35,36^, there is still different levels of annotations (from lipid class to stereoisomers) and different ontologies among the databases in use.

In these publications, the diversity of outcome, related to cardiometabolic risk was found to be important. Moreover, the lack of description regarding either other MetS criteria or characteristics of controls, together with the absence of additional phenotypic data (*e.g*. physical activity, nutrition) in some publications, prevented us from including them in this review. For example, plasma metabolite concentrations are known to be highly influenced by physical activity and/or microbiota^37–39^ and plasma phospholipids were proposed to be indicative of both food habits and metabolic changes^40^. It has been recognized that publication of all the metadata (data about the samples) along with the metabolomic data is a good practice to assess the quality of the models and the drawn conclusions. Despite the existing data repositories in the field (MetaboLights^41^, Metabolomics Workbench^42^) and available guidelines provided by the metabolomics standards initiative (MSI)^43,44^, such good practice is still quite rare.

Despite these limitations, this review highlights the importance of amino acids and TGs, which have both been described as MetS biomarkers and associated with each of the five clinical MetS criteria. In fact, previously alterations of serum amino acids have been reported in the development of overweight, obesity, and insulin resistance^45,46^. Increased TG levels have also been linked to obesity and insulin resistance^47^, but even if associations with hypertension and hypertension risk were shown, the involved mechanisms remain to be explored^48^.

### The glycemic component: towards T2D

Among all the MetS criteria, elevated fasting blood glucose was by far the most studied phenotype using metabolomics/lipidomics, because of its direct link with T2D. Studies on dysglycemia have been among the main drivers in this research field using global metabolomic approaches for biomarker discovery and validation. This review allows first to get an overview of the publications considering this specific component among a whole set of metabolic risks, which is of great interest, in the context of systems approaches. In particular, it highlights the interest of profiling both amino acids, lipids and carbohydrates to decipher the complex interplay between obesity and diabetes, as previously discussed^25^. In addition, it allows identifying specific metabolites of interest such as isoleucine, α-hydroxybutyrate, and ether phosphaditylcholine (PC) species to monitor disease progression in the context of metabolic disorders. In fact, although little studied, ether PC species are part of an overlapping lipid profile between diabetes and hypertension^49^. Further, this review illustrates the use of metabolomics as a powerful tool for the identification of relevant pattern of hundreds of detected metabolites that could be used to predict future development of T2D. However, metabolic profiles acquired with semi- or non-targeted approaches are complex and required dedicated variable selection to build powerful predictive models of specific prediabetic phenotypes^50^. As the analysis of data is one of the most challenging steps in the metabolomics approach due to high data dimensionality and limited number of samples, recommendations as well as appropriate statistical workflows have been proposed. They often include a combination of univariate and multivariate analyses and highlighted the importance of feature/variable selection and external validation to minimize the risk of overfitting^51,52^. In most publications included in the present review, statistical approaches were not described in detail and limited to univariate analyses, which are the most commonly used due to their easiness of interpretation. However, in the context of metabolomics/lipidomics, multivariate methods are of great relevance as they make use of all variables simultaneously and deal with the relationship between variables, reflecting orchestrated biological processes^53^.

### Limitations and recommendations for further studies

An important limitation concerning this review is the intrinsic issue of selecting a targeted metabolomic or lipidomic approach or interpreting the resulting data in connection with the study design and the phenotypes of interest. Such a strategy can lead to difficulties in interpretation due to missing acquired data on relevant pathways from this context. In addition, around 60% of the selected studies were using only metabolomics, which is probably the best compromise when using a single approach, as it also allows detecting the most polar lipid families. However, considering the multifaceted physiopathology of MetS, it is of great interest to consider applying a more comprehensive strategy using both untargeted metabolomics and lipidomics to cover the large diversity of potential modulated metabolites in biofluids. This combination is still rare (only three studies in the present review) most probably because of costs, expertise, and complexity of data analytical treatment.

A second limitation concerns methods both for data production and treatment. Regarding sample preparation and analytical methods, experimental conditions were very heterogeneous, making comparison between studies challenging. Moreover, in the selected articles, even if confounding factors have been often considered in study designs, data description and analysis of these potentially interacting factors were frequently lacking. Such biases have often been identified and statistical approaches have been developed to avoid false discoveries in metabolomics^52^. Beyond this aspect, multiple ontologies used to describe metabolites/lipids^54^ and the semi quantitative property of most of the analytical methods, are still major bottlenecks of the field.

Despite these limitations, it is now recognized that metabolomics is a powerful tool allowing metabolic stratification of patients and prognosis^55^. Indeed a metabolic signature would lead to a molecular definition of MetS^56^, as exemplified by Wiklung et *al*.^57^ and Pujos-Guillot et *al*.^58^. Clinically speaking, the interest of subtyping MetS has been shown since the prevalence and risk for further cardiovascular disease and T2D is associated with different combinations of its components^15^. More recently, Sperling et *al*.^59^ highlighted the need of identifying subtypes of MetS on the basis of pathophysiology, as well as studying the evolution of its stages for a more efficient prevention and therapy. In this context, metabolomic and lipidomic signatures are suitable systems approaches not only to identify biomarkers of sub-phenotypes but also for hypothesis generation of the underlying pathogenic mechanisms.

## Conclusion

The present review indicates that relatively few articles have been published so far on MetS biomarkers identification using metabolomics and lipidomics in adults. Unfortunately, due to many limitations previously highlighted, it is difficult to compare conclusions from the available data. Moreover, individual MetS clinical components were not specifically investigated, despite the fact that metabolomics/lipidomics are recognized as being powerful phenotyping tools in chronic metabolic diseases. Since studies on T2D have been among the main drivers in this research field using these global approaches for biomarker discovery and validation, it can be concluded that metabolomics and lipidomics signatures could be the strategy of choice for a deeper investigation and characterization of MetS and its sub-phenotypes. Considering future research, a number of key recommendations can be made. First, untargeted methods must be performed using multiplatform approaches for a wide detection of metabolite diversity enabling new biomarker discovery. Second, the complexity of metabolomic/lipidomic data has to be investigated using dedicated univariate and multivariate statistics and data reporting has to follow the FAIR principle^60^, concerning both population characteristics and marker metadata. This issue is crucial to ensure the reliability, validity and inter-comparability of experimental results. Such effort should allow transferring knowledge from basic research to clinical practices.

## Materials and Methods

### Methodology for review of published literature

The systematic review of the literature was performed according to the Preferred Reporting Items for Systematic Reviews and Meta-Analyses (PRISMA) guidelines for conducting systematic reviews^61^.

A specific request was made through several bibliographic electronic databases in August 2019. All databases were chosen in line with the application field studied in the review, namely health research and biology, and five were retained: MEDLINE (from 1946 onwards), EMBASE (from 1974 onwards), EMB Review (from 1991 onwards), CINHAL Complete (from 1937 onwards) and PubMed. To ensure that information collected was complete, the request was also performed on grey literature ((CADTH, Clinical Trials, National Guideline Clearing House, National Institute for Health and Care Excellence (NICE), MedNar, Google Scholar and Open Grey). The request combined words and expressions for three conceptual groups: “Metabolomics/lipidomics”, “Metabolic Syndrome” and “metabolites/biomarkers” (Supplemental Material 1). For each database, words and expressions from controlled vocabulary (MeSH, EMTREE and others) and free-text searching were used. Snowballing techniques and Handsearching was also used to identify other references. Duplicate publications were deleted.

### Study selection and data extraction

Initially, titles and abstracts were screened by two authors using the following inclusion and exclusion criteria: (1) articles had to be published in English; (2) publications had to contain original data, therefore reviews, book chapters, and editorials were excluded; (3) studies on non-human models (*e.g*. animals, plants, cells) were excluded; human studies were restricted to case/control, observational, and prospective designs; intervention studies were excluded. Finally, population was restricted to adult/aging Caucasian subjects; thus articles on children, adolescents or pregnant women were excluded; (4) the primary outcome had to be the MetS and/or its components, including T2D, and (5) articles referring to genetic/transcriptomic markers or proteomics were also excluded. These two authors resolved disagreements. To determine publication relevance, three authors independently screened all titles and abstracts to assess their eligibility against the following more restrictive criteria: Eligible publications in the review had to include a minimum of 20 subjects per group and available clinical data regarding the MetS criteria: fasting glucose, TG, HDL-C concentrations, waist circumference, systolic and diastolic blood pressures. Concerning the number of subjects considered as minimum per study, it is generally admitted that 30 subjects is a limit to be able to perform common methods in statistics, in relation to a normal distribution. Moreover, because of the diversity/complexity of the MetS metabolic phenotypes, influenced by numerous factors (gender, age, diet…), taking a population of 40 subjects (i.e. 20 subjects per group for a case/control study) was considered as a minimum requirement. Disagreements in abstracts inclusion were resolved after consensual decision involving a fourth author.

Pertinent data from papers were then extracted, including, author names, publication year, study population and design, number of subjects, gender/sex, baseline clinical characteristics and main outcome. The experimental measures were collected regarding the nature of the biological samples, the analytical approach and techniques, and information regarding statistical methods and covariates when relevant. The results were analysed and compiled by biochemical family including significantly modulated metabolites (p-value < 0.05), metabolite listings with levels of change according to the outcome and/or MetS clinical criteria. Finally, results from different studies were compared using Venn diagrams^62^ to obtain a more synthetic view.

### Ethics statement

This article does not contain any studies with human or animal subjects performed by any of the authors.

## Supplementary information

Supplementary Information
